# COVID-19 Pneumonia Diagnosis Using a Simple 2D Deep Learning Framework With a Single Chest CT Image: Model Development and Validation

**DOI:** 10.2196/19569

**Published:** 2020-06-29

**Authors:** Hoon Ko, Heewon Chung, Wu Seong Kang, Kyung Won Kim, Youngbin Shin, Seung Ji Kang, Jae Hoon Lee, Young Jun Kim, Nan Yeol Kim, Hyunseok Jung, Jinseok Lee

**Affiliations:** 1 Department of Biomedical Engineering Wonkwang University College of Medicine Iksan-si Republic of Korea; 2 Department of Trauma Surgery Wonkwang University Hospital Iksan-si Republic of Korea; 3 Department of Radiology and Research Institute of Radiology Asan Image Metrics, Clinical Trial Center, Asan Medical Center University of Ulsan College of Medicine Seoul Republic of Korea; 4 Department of Internal Medicine Chonnam National University Medical School Gwangju-si Republic of Korea; 5 Department of Internal Medicine Wonkwang University Hospital Iksan-si Republic of Korea; 6 Department of Radiology Wonkwang University Hospital Iksan-si Republic of Korea

**Keywords:** COVID-19, deep learning, convolutional neural networks, transfer learning, chest CT, CT, neural network, pneumonia, artificial intelligence, diagnosis, pneumonia, scan

## Abstract

**Background:**

Coronavirus disease (COVID-19) has spread explosively worldwide since the beginning of 2020. According to a multinational consensus statement from the Fleischner Society, computed tomography (CT) is a relevant screening tool due to its higher sensitivity for detecting early pneumonic changes. However, physicians are extremely occupied fighting COVID-19 in this era of worldwide crisis. Thus, it is crucial to accelerate the development of an artificial intelligence (AI) diagnostic tool to support physicians.

**Objective:**

We aimed to rapidly develop an AI technique to diagnose COVID-19 pneumonia in CT images and differentiate it from non–COVID-19 pneumonia and nonpneumonia diseases.

**Methods:**

A simple 2D deep learning framework, named the fast-track COVID-19 classification network (FCONet), was developed to diagnose COVID-19 pneumonia based on a single chest CT image. FCONet was developed by transfer learning using one of four state-of-the-art pretrained deep learning models (VGG16, ResNet-50, Inception-v3, or Xception) as a backbone. For training and testing of FCONet, we collected 3993 chest CT images of patients with COVID-19 pneumonia, other pneumonia, and nonpneumonia diseases from Wonkwang University Hospital, Chonnam National University Hospital, and the Italian Society of Medical and Interventional Radiology public database. These CT images were split into a training set and a testing set at a ratio of 8:2. For the testing data set, the diagnostic performance of the four pretrained FCONet models to diagnose COVID-19 pneumonia was compared. In addition, we tested the FCONet models on an external testing data set extracted from embedded low-quality chest CT images of COVID-19 pneumonia in recently published papers.

**Results:**

Among the four pretrained models of FCONet, ResNet-50 showed excellent diagnostic performance (sensitivity 99.58%, specificity 100.00%, and accuracy 99.87%) and outperformed the other three pretrained models in the testing data set. In the additional external testing data set using low-quality CT images, the detection accuracy of the ResNet-50 model was the highest (96.97%), followed by Xception, Inception-v3, and VGG16 (90.71%, 89.38%, and 87.12%, respectively).

**Conclusions:**

FCONet, a simple 2D deep learning framework based on a single chest CT image, provides excellent diagnostic performance in detecting COVID-19 pneumonia. Based on our testing data set, the FCONet model based on ResNet-50 appears to be the best model, as it outperformed other FCONet models based on VGG16, Xception, and Inception-v3.

## Introduction

The coronavirus disease (COVID-19) pandemic is currently a global health crisis; more than 1,700,000 cases had been confirmed worldwide and more than 100,000 deaths had occurred at the time of writing this paper [1]. COVID-19, an infection caused by severe acute respiratory syndrome coronavirus 2 (SARS-CoV-2), is highly contagious and has spread rapidly worldwide. In severe cases, COVID-19 can lead to acute respiratory distress, multiple organ failure, and eventually death. Countries are racing to slow the spread of the virus by testing and treating patients in the early stage as well as quarantining people who are at high risk of exposure due to close contact with patients with confirmed infection. In addition, early diagnosis and aggressive treatment are crucial to saving the lives of patients with confirmed infection [2].

COVID-19 is typically confirmed by viral nucleic acid detection using reverse transcription–polymerase chain reaction (RT-PCR) [3]. However, the sensitivity of RT-PCR may not be sufficiently high; it ranges from 37% to 71% according to early reports [4-6]. Thus, RT-PCR can afford a substantial number of false negative results due to inadequate specimen collection, improper extraction of nucleic acid from the specimen, or collection at a too-early stage of infection. A chest computed tomography (CT) scan can be used as an important tool to diagnose COVID-19 in cases with false negative results by RT-PCR [6-9].

Recently, a multinational consensus statement from the Fleischner Society was issued to guide chest imaging during the COVID-19 pandemic in different clinical settings [6]. According to this consensus statement, in a setting such as South Korea, where detecting patients at an early stage and isolating all patients and people with high risk of exposure is essential, CT is a relevant screening tool due to its greater sensitivity for detecting early pneumonic changes. CT can also contribute to the management and triage of the disease by detecting severe cases. In addition, chest CT is noninvasive and is easy to perform in an equipped facility.

However, radiologic diagnostic support is not maintained 24 hours per day in many institutions [10]. In addition, CT may show similar imaging features between COVID-19 and other types of pneumonia, thus hampering correct diagnosis by radiologists. Indeed, in a study that evaluated radiologists’ performance in differentiating COVID-19 from other viral pneumonia, the median values and ranges of sensitivity and specificity were 83% (67%-97%) and 96.5% (7%-100%), respectively [11].

The use of artificial intelligence (AI) may help overcome these issues, as AI can help maintain diagnostic radiology support in real time and with increased sensitivity [8,12]. In this era of worldwide crisis, it is crucial to accelerate the development of AI techniques to detect COVID-19 and to differentiate it from non–COVID-19 pneumonia and nonpneumonia diseases in CT images. Therefore, we aimed to rapidly develop an AI technique using all available CT images from our institution as well as publicly available data.

## Methods

### Data Sets and Imaging Protocol

This study was approved by the institutional review boards of Wonkwang University Hospital (WKUH) and Chonnam National University Hospital (CNUH). Informed consent was waived. Table 1 summarizes the training, testing, and additional validation data sets. In this study, we initially collected data from 3993 chest CT images, which were categorized into COVID-19, other pneumonia, and nonpneumonia disease groups.

For the COVID-19 data group, we used a total of 1194 chest CT images: 673 chest CT images (56.3%, from 13 patients) from CNUH, 421 images (35.3%, from 7 patients) from WKUH, and 100 images (83.8%, 60 patients) from the Italian Society of Medical and Interventional Radiology (SIRM) public database [13]. The 20 patients from CNUH and WKUH included 9 male patients and 11 female patients, with an average age of 59.6 years (SD 17.2). Regarding the COVID-19 data from WKUH and CNUH, all the patients with COVID-19 tested positive for the virus by RT-PCR viral detection, and the CT images were acquired between December 31, 2019 and March 25, 2020. The median period from symptom onset to the first chest CT examination was 8 days (range 2-20 days). The most common symptoms were fever (75%) and myalgia (30%). In addition, according to previous studies related to COVID-19 by Zhao’s group [14] from January 19 and March 25, 2020, 264 low-quality chest CT images were used as additional testing data. In summary, 1194 COVID-19 images (80 patients) from WKUH, CNUH, and SRIM were split into the training data set (955 images, 80.0%) and testing data set (239 images, 20.0%). For the additional testing, 264 COVID-19 images (264 patients) from the low-quality image data set were used.

**Table 1:** Summary of the training, testing, and additional testing data sets (N=4257).

Data type, data source, and data group			Training images, n (%)		Testing images, n (%)	
**Training and testing data**						
	**WKUH^a^**					
		COVID-19 pneumonia (n=421)		337 (80.0)		84 (20.0)
		Other pneumonia (n=1357)		1086 (80.0)		271 (20.0)
		Nonpneumonia and normal lung (n=998)		798 (80.0)		200 (20.0)
		Lung cancer (n=444)		355 (80.0)		89 (20.0)
	**CNUH^b^**					
		COVID-19 pneumonia (n=673)		538 (80.0)		135 (20.0)
	**SIRM^c^**					
		COVID-19 pneumonia (n=100)		80 (80.0)		20 (20.0)
**Additional external testing data**						
	**Low-quality CT images from papers**					
		COVID-19^d^ pneumonia (n=264)		0 (0.0)		264 (100.0)

^a^WKUH: Wonkwang University Hospital.

^b^CNUH: Chonnam National University Hospital.

^C^SIRM: Italian Society of Medical and Interventional Radiology.

^d^COVID-19: coronavirus disease.

For the other pneumonia data group, we selected 1357 chest CT images from 100 patients diagnosed with non–COVID-19 pneumonia at WKUH between September 1, 2019, and March 30, 2020. The average age of this group was 62.5 years (SD 17.2), with 68 male and 32 female patients. For the nonpneumonia data group, we also selected 1442 chest CT images from 126 patients who had no lung parenchymal disease or lung cancers at WKUH between January 2009 and December 2014. The average age of these patients was 47 years (SD 17), with 63 male patients (721/1442 images, 50.0%) and 63 female patients (721/1442 images, 50.0%). The patient demographic statistics of the COVID-19 and other pneumonia groups are summarized in Table 2. In this table, other pneumonia (not COVID-19) was categorized into two different types based on clinical characteristics: 68 cases of community-acquired pneumonia (onset 48 hours before hospital admission) and 32 cases of hospital-acquired pneumonia (onset 48-72 hours after hospital admission). Of these other pneumonia patients, 24/100 (24.0%) received laboratory confirmation of the etiology, 21 (21.0%) were confirmed to be bacterial culture positive, 3 (3.0%) were viral influenza positive by RT-PCR, and 76 (76.0%) were negative. Regarding the imaging protocols, each volumetric examination contained approximately 51 to 1094 CT images, with varying slice thicknesses from 0.5 millimeters to 3 mm. The reconstruction matrix was 512×512 pixels, with in-plane pixel spatial resolution from 0.29×0.29 to 0.98×0.98 square millimeters.

The data from WKUH, CNUH, and SIRM were randomly split with a ratio of 8:2 into a training set and a testing set, respectively, in a stratified fashion. In addition, the data for each group (WKUH, CNUH, and SIRM) were spread over different splits with a ratio of 8:2. The training data set was then further separated into sets used for training the model (80% of the training set) and for internal validation (20% of the training set). The testing set was used only for independent testing of the developed models and was never used for training the model or for internal validation. Furthermore, we tested the trained model with the additional external validation data set of low-quality images to evaluate the external generalizability of the model.

**Table 2:** Demographic data of patients with COVID-19 and other pneumonia.

Characteristic		COVID-19^a^ pneumonia (n=20)	Other pneumonia (n=100)	*P* value
Age (years), mean (SD)		59.6 (17.2)	60.1 (17.1)	.91
Male sex, n (%)		9 (45.0)	68 (68.0)	.12
Community-acquired pneumonia, n (%)		20 (100.0)	68 (68.0)	.007
Hospital-acquired pneumonia, n (%)		0 (0.0)	32 (32.0)	
**Microbiological study, n (%)**				
	COVID-19 positive (RT-PCR^b^)	20 (100.0)	0 (0.0)	<.001
	Other virus positive (influenza)	0 (0.0)	3 (3.0)	
	Bacterial culture positive	0 (0.0)	21 (21.0)	
	Unknown	0 (0.0)	76 (76.0)	
**Human radiologist's diagnosis, n (%)**				
	Atypical pneumonia or viral pneumonia	20 (100.0)	15 (15.0)	N/A^c^
	Pneumonia	0 (0.0)	77 (77.0)	
	Aspiration pneumonia	0 (0.0)	26 (26.0)	
	Necrotizing pneumonia	0 (0.0)	5 (5.0)	
	Tuberculosis	0 (0.0)	5 (5.0)	
	Empyema	0 (0.0)	3 (3.0)	
	Emphysema	0 (0.0)	9 (9.0)	
	Bronchiectasis	0 (0.0)	4 (4.0)	
	Interstitial lung disease	0 (0.0)	1 (1.0)	

^a^COVID-19: coronavirus disease.

^b^RT-PCR: reverse transcription–polymerase chain reaction.

^c^N/A: not applicable.

### Preprocessing

For the data acquired from WKUH and CNUH, we converted Digital Imaging and Communications in Medicine (DICOM) images to one-channel grayscale PNG images to standardize the image file format, as the images in the low-quality image data set were in PNG format. To minimize the information loss, we first displayed the DICOM images using a lung window with a 1500 Hounsfield unit window width and a –600 HU window level [15,16] and converted the images to PNG format. Subsequently, we rescaled the images to a size of 256×256 pixels and normalized the pixel values to a range between 0 and 1. All of the converted PNG format images were confirmed by three radiologists to determine any loss of image information related to pulmonary diseases. For the data from SIRM, the original JPEG format was also reformatted to the PNG format, and the images were rescaled and normalized in the same manner. For the low-quality image data set, we also rescaled and normalized the images. In this study, no further preprocessing such as lung segmentation was performed.

### Image Augmentation

To reduce overfitting of the training image data, we employed two distinct forms of data augmentation: image rotation and zoom. In the data augmentation method for the rotation, angles of rotation between –10° and 10° were randomly selected. Regarding the zoom, the range was randomly selected between 90% (zoom-in) and 110% (zoom-out). Either rotation or zoom was randomly selected 10 times for each training image. By applying data augmentation, we increased the number of images in the training data set to 31,940. Table 3 shows the number of augmented images for training in each group.

**Table 3:** Augmented images for training in each group (N=31,940).

Data source and group		Augmented images for training, n (%)
**WKUHx^a^**		
	COVID-19^b^ pneumonia	3370 (10.6)
	Other pneumonia	10,860 (34.0)
	Nonpneumonia and normal lung	7890 (24.7)
	Lung cancer	3550 (11.1)
**CNUH^c^**		
	COVID-19 pneumonia	5380 (16.8)
**SIRM^d^**		
	COVID-19 pneumonia	800 (2.5)

^a^WKUH: Wonkwang University Hospital.

^b^COVID-19: coronavirus disease

^c^CNUH: Chonnam National University Hospital.

^d^SIRM: Italian Society of Medical and Interventional Radiology.

### The Fast-Track COVID-19 Classification Network for COVID-19 Classification

**Figure 1:**
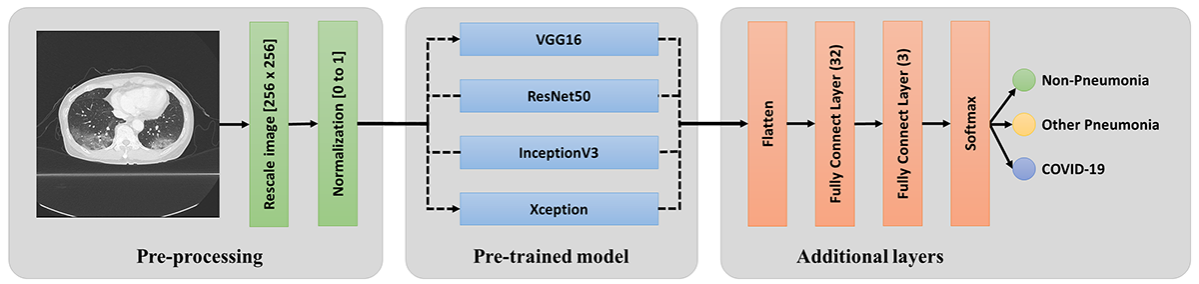
Scheme of FCONet, a 2D deep learning framework based on a single chest CT image for the classification of COVID-19 pneumonia, other pneumonia, and non-pneumonia. COVID-19: coronavirus disease.

We developed a simple 2D deep learning framework based on a single chest CT image for the classification of COVID-19 pneumonia, other pneumonia, and nonpneumonia, named the fast-track COVID-19 classification network (FCONet; Figure 1). FCONet was developed by transfer learning based on one of the following four pretrained convolutional neural network (CNN) models as a backbone: VGG16 [17], ResNet-50 [18], Inception-v3 [19], and Xception [20]. Transfer learning is a popular method in computer vision because it enables an accurate model to be built in a short time [21]. With transfer learning, instead of starting the learning process from an optimal model search, one can start it from patterns that were learned when solving a different problem. To minimize the training time, we initially used the predefined weights for each CNN architecture, which were further updated through the learning process of classification of COVID-19 pneumonia, other pneumonia, and nonpneumonia.

#### Input Layer

After the simple preprocessing stage, in the *input layer*, we arranged three channels (256×256×3 pixels) by copying the one-channel normalized image. The three-channel images were fed into *the pretrained model layers*.

#### Pretrained Model Layers

A pretrained model is a model that was trained on a large benchmark data set to solve a similar problem to the one we want to solve. In the *pretrained model layers*, we included one of the four pretrained models (VGG16, ResNet-50, Inception-v3, and Xception). Each model comprises two parts: a convolutional base and a classifier. The convolutional base is composed of a stack of convolutional and pooling layers to generate features from the images. The role of the classifier is to categorize the image based on the extracted features. In our pretrained model layers, we retained the convolutional base and removed the classifier, which was replaced by another classifier for COVID-19, other pneumonia, or nonpneumonia.

#### Additional Layers

The activations from the *pretrained model layers* were fed into the *additional layers*. The layers acted as classifiers for COVID-19 pneumonia, other pneumonia, and nonpneumonia. In the additional layers, we first flattened the activations and connected two fully connected layers; one of the layers consisted of 32 nodes, and the other consisted of three nodes. Subsequently, the three activations from the second fully connected layer were fed into a SoftMax layer, which provided the probability for each of COVID-19, other pneumonia, and nonpneumonia.

#### Implementation

We implemented FCONet using the TensorFlow package, which provides a Python application programming interface (API) for tensor manipulation. We also used Keras as the official front end of TensorFlow. We trained the models with the Adam optimizer [22] and the categorical cross-entropy cost function with a learning rate of 0.0001 and a batch size of 32 on a GeForce GTX 1080 Ti graphics processing unit (NVIDIA). For the performance evaluation, 5-fold cross-validation was performed to confirm the generalization ability. The training data set (N=31,940) was randomly shuffled and divided into five equal groups in a stratified manner. Subsequently, four groups were selected to train the model, and the remaining group was used for validation. This process was repeated five times by shifting the internal validation group. Next, we averaged the mean validation costs of the five internal validation groups according to each epoch and found the optimal epoch that provides the lowest validation cost. Then, we retrained the model using the entire training data set with the optimal epoch. The testing data set was evaluated only after the model was completely trained using the training data set. This holdout method provides an unbiased evaluation of the final model by avoiding overfitting to the training data set.

### Performance Evaluation and Statistical Evaluation

For each of the different four pretrained models (VGG16, ResNet-50, Inception-v3, and Xception) in FCONet, we evaluated the classification performance based on sensitivity, specificity, and accuracy. More specifically, we calculated true positives (TP), false positives (FP), true negatives (TN), and false negatives (FN) based on the groups of COVID-19 pneumonia, other pneumonia, and nonpneumonia. For each group, we expressed measure metrics with the subscripts *covid* for COVID-19, *other* for other pneumonia, and *none* for nonpneumonia, as

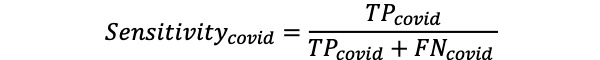

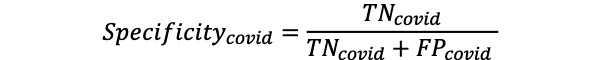

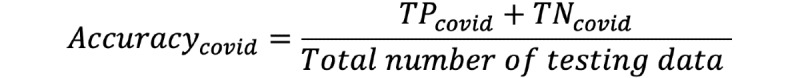







where *TP_covid_* is the number of COVID-19 testing data correctly classified as COVID-19, *TN_covid_* is the number of non–COVID-19 testing data correctly classified as non–COVID-19, *FP_covid_* is the number of non–COVID-19 testing data misclassified as COVID-19, and *FN_covid_* is the number of COVID testing data misclassified as non–COVID-19. Here, non–COVID-19 testing data include other pneumonia and nonpneumonia. Note that the same calculations were applied to the other pneumonia and nonpneumonia testing data as

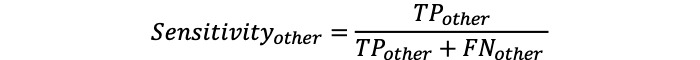

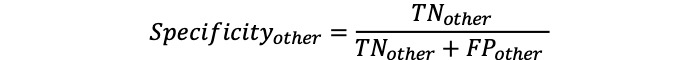

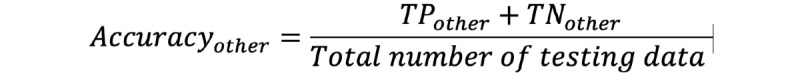

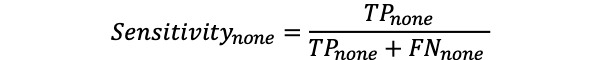

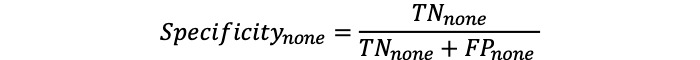

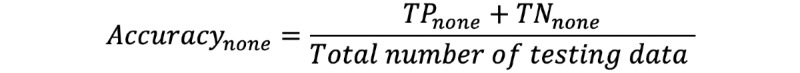













We also plotted the receiver operating characteristic (ROC) curve and calculated the area under the curve (AUC) for each of the four different models. Additionally, statistical analysis was performed using MATLAB (R2013b). Analysis of variance (ANOVA) was used to compare differences among COVID-19 pneumonia, non–COVID-19 pneumonia, and nonpneumonia groups. A *P* value less than .001 was considered to indicate statistical significance.

## Results

The performance of the FCONet models based on the four pretrained models in the classification of COVID-19 pneumonia, other pneumonia, and nonpneumonia is summarized in Table 4. We compared the metric values of sensitivity (%), specificity (%), and accuracy (%) as well as the AUCs of the four FCONet models based on VGG16, ResNet-50, Inception-v3, and Xception. Based on the testing data, the FCONet models based on ResNet-50, VGG16, and Xception showed excellent classification performance; all these models provided AUC values ranging from 0.99 to 1.00. More specifically, with ResNet-50, the sensitivity, specificity, and accuracy for classifying COVID-19 pneumonia were 99.58%, 100%, and 99.87%, respectively. With VGG16, the sensitivity, specificity, and accuracy for classifying COVID-19 pneumonia were 100%, 99.64%, and 99.75%, respectively. With Xception, the sensitivity, specificity, and accuracy for COVID-19 pneumonia classification were 97.91%, 99.29%, and 98.87%, respectively. For other pneumonia and nonpneumonia, the sensitivity, specificity, and accuracy ranged from 97% to 100% when ResNet-50, VGG16, or Xception was used as the backbone in FCONet. On the other hand, Inception-v3–based FCONet provided relatively low sensitivity, specificity, and accuracy values for all groups of COVID-19 pneumonia, other pneumonia, and nonpneumonia (*P*<.001).

**Table 4:** Performance of the FCONet frameworks based on the four pretrained models on the testing data set.

Model and data group		Sensitivity, %	Specificity, %	Accuracy, %	AUC^a^		*P* value
**ResNet-50**						<.001	
	COVID-19^b^ pneumonia	99.58	100.00	99.87	1.00		
	Other pneumonia	97.42	99.81	99.00	0.99		
	Nonpneumonia	100.00	98.63	99.12	0.99		
**VGG16**						<.001	
	COVID-19 pneumonia	100.00	99.64	99.75	1.00		
	Other pneumonia	100.00	99.81	99.87	0.99		
	Nonpneumonia	100.00	99.80	99.87	0.99		
**Xception**						<.001	
	COVID-19 pneumonia	97.91	99.29	98.87	0.99		
	Other pneumonia	98.52	99.05	98.87	0.99		
	Nonpneumonia	100.00	100.00	100.00	1.00		
**Inception-v3**						<.001	
	COVID-19 pneumonia	88.28	97.68	94.87	0.97		
	Other pneumonia	94.10	95.83	95.24	0.98		
	Nonpneumonia	98.27	97.25	97.62	0.99		

^a^AUC: area under the curve.

^b^COVID-19: coronavirus disease.

**Figure 2:**
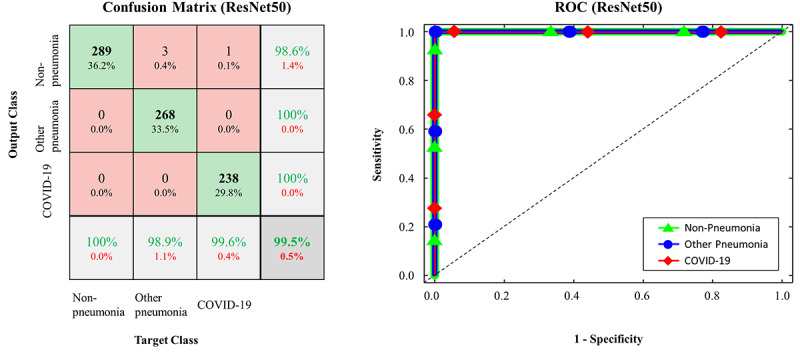
Confusion matrix and ROC curve in FCONet using ResNet-50; COVID-19: coronavirus disease; ROC: receiver operating characteristic.

**Figure 3:**
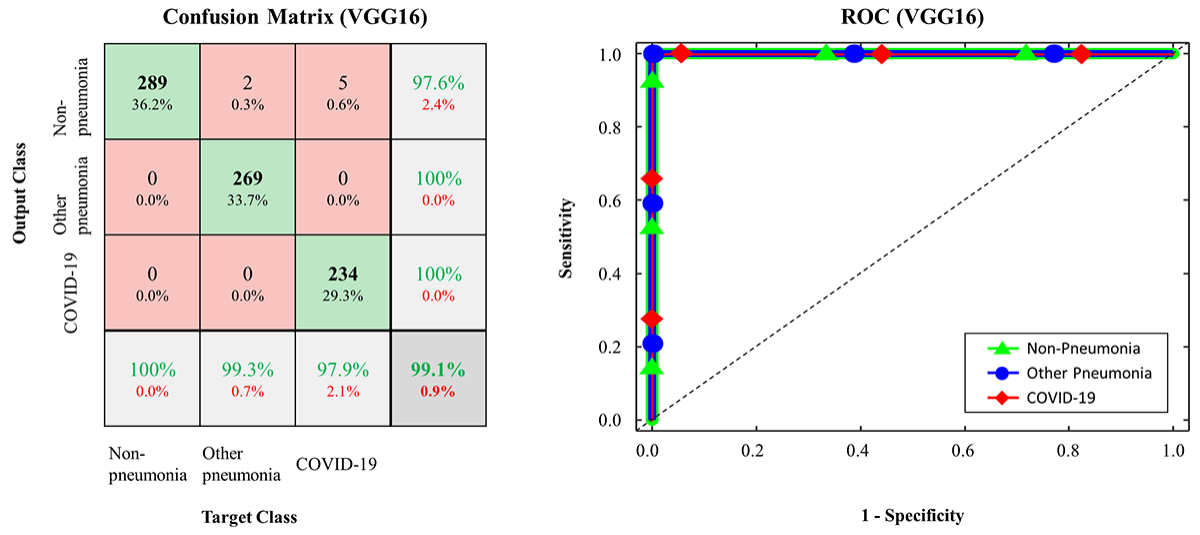
Confusion matrix and ROC curve in FCONet using VGG16; COVID-19: coronavirus disease; ROC: receiver operating characteristic.

**Figure 4:**
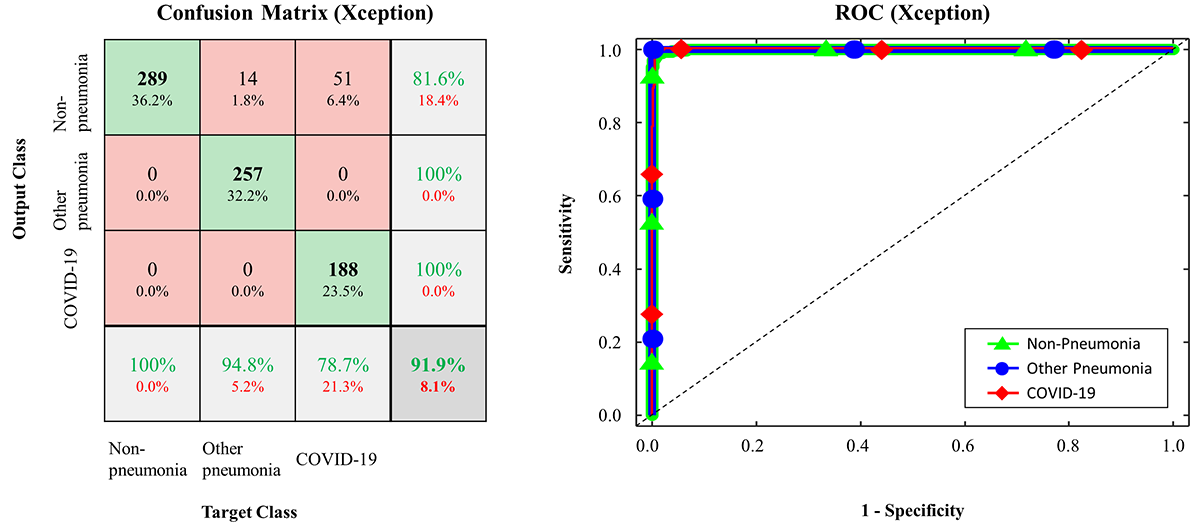
Confusion matrix and ROC curve in FCONet using Xception; COVID-19: coronavirus disease; ROC: receiver operating characteristic.

**Figure 5:**
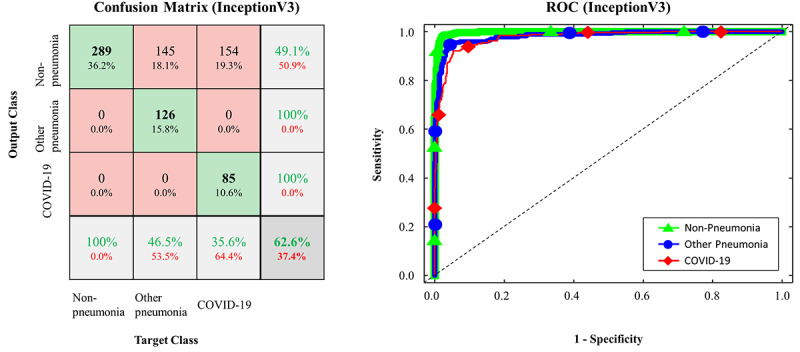
Confusion matrix and ROC curve in FCONet using Inception-v3; COVID-19: coronavirus disease; ROC: receiver operating characteristic.

The confusion matrices and ROC curves for the pretrained models on the testing data set are presented in Figures 2-5. More specifically, ResNet-50 exhibited *TP_covid_*, *TP_other_*, and *TP_none_* of 238/239, 268/271, and 289/289, respectively (Figure 2). VGG16 exhibited *TP_covid_*, *TP_other_*, and *TP_none_* of 234/239, 269/271, and 289/289, respectively (Figure 3). Xception exhibited *TP_covid_*, *TP_other_*, and *TP_none_* of 188/239, 257/271, and 289/289, respectively (Figure 4). Inception-v3 exhibited *TP_covid_*, *TP_other_*, and *TP_none_* of 211/239, 255/271, and 284/289, respectively (Figure 5). For the three models of ResNet-50, VGG16, and Xception, the values of AUC were very close to 1 because the predicted probability values were provided as values close to 1 for correct labeling and values close to 0 for incorrect labeling.

On the additional external validation data set, which comprised low-quality CT images of COVID-19 pneumonia embedded in recently published papers, the detection accuracy of ResNet-50 was the highest with 96.97%, followed by Xception (90.71%), Inception-v3 (89.38%), and VGG16 (87.12%) (Table 5).

**Table 5:** Performance of each deep learning model on the additional external validation data set of COVID-19 pneumonia images.

Model	Detection accuracy, %
ResNet-50	96.97
VGG16	87.12
Xception	90.71
Inception-v3	89.38

**Figure 6:**
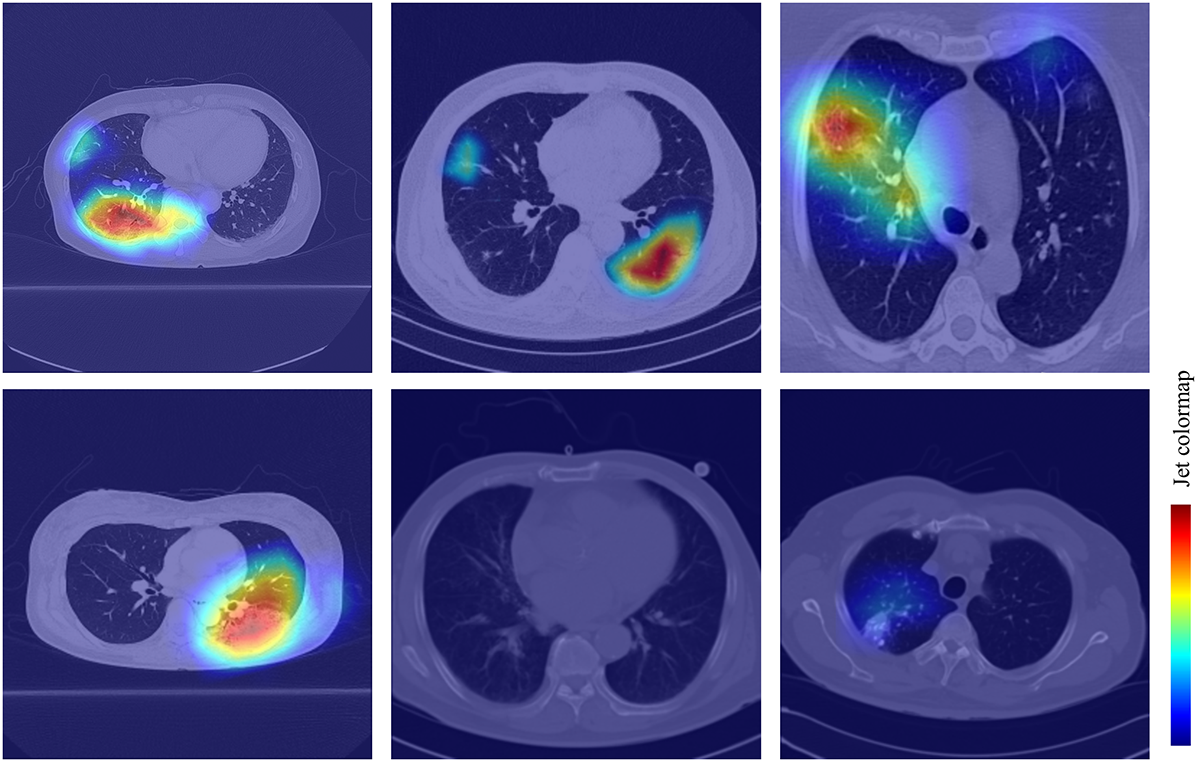
Confusion matrice and ROC curve in FCONet using VGG16; COVID-19: coronavirus disease; ROC: receiver operating characteristic.

To improve the interpretability of our model, we used the gradient-weighted class activation mapping (Grad-CAM) method [23] to visualize the important regions leading to the decision of FCONet. The model fully generates this localization map without the mapping annotation. The heatmaps (Figure 6) show the suspected regions for the examples of COVID-19, other pneumonia, and nonpneumonia. The heatmaps are standard jet colormaps and are overlapped on the original image, where red color highlights the activation region associated with the predicted class. More specifically, for the COVID-19 image group, the heatmap strongly indicated the suspected regions, as shown in examples from WKUH (Figure 6, top left), CNUH (Figure 6, top middle) and SIRM (Figure 6, top right). For the other pneumonia image groups, the heatmap demonstrated some suspected regions inside the lung area; the intensity was lower than that of the regions in the COVID-19 image group (Figure 6, bottom left). For the healthy image group, there was no heatmap corresponding to the suspected regions (Figure 6, bottom middle). For the lung cancer images, the heatmap indicated some suspected regions inside the lung area; however, the intensity was also lower than that of the regions in the COVID-19 pneumonia group (Figure 6, bottom right).

To test the generalizability of our proposed framework, we also trained and tested the models based on institutional data split for COVID-19 data: training data from CNUH and SIRM and tested data from WKUH. Because the COVID-19 data were split with a ratio of 65:35 (773 training data and 421 testing data for COVID-19), the other non–COVID-19 data were randomly split with the same ratio in a stratified fashion. Table 6 summarizes the performance of the FCONet framework. With ResNet-50, the sensitivity, specificity and accuracy for classifying COVID-19 pneumonia were 97.39%, 99.64% and 98.67%, respectively (*P*<.001). With VGG16, the sensitivity, specificity, and accuracy for classifying COVID-19 pneumonia were 97.15%, 99.64% and 98.57%, respectively (*P*<.001). With Xception, the sensitivity, specificity, and accuracy for classifying COVID-19 pneumonia were 90.50%, 94.82% and 92.97%, respectively (*P*<.001). With Inception-v3, the sensitivity, specificity, and accuracy for classifying COVID-19 pneumonia were 74.58%, 99.46% and 88.79%, respectively (*P*<.001). These results show that the FCONet framework can classify COVID-19 regardless of the data split approach.

**Table 6:** Performance of the FCONet framework based on institutional data split for COVID-19 data.

Model and data group		Sensitivity, %	Specificity, %	Accuracy, %	AUC^a^		*P* value
**ResNet-50**						<.001	
	COVID-19 pneumonia	97.39	99.64	98.67	0.99		
	Other pneumonia	99.26	98.45	98.637	0.99		
	Nonpneumonia	100	100	100	1.0		
**VGG16**						<.001	
	COVID-19 pneumonia	97.15	99.64	98.57	0.99		
	Other pneumonia	99.26	98.31	98.57	0.99		
	Nonpneumonia	100	100	100	1.0		
**Xception**						<.001	
	COVID-19 pneumonia	90.50	94.82	92.97	0.98		
	Other pneumonia	89.30	94.37	92.97	0.98		
	Nonpneumonia	100	100	100	1.0		
**Inception-v3**						<.001	
	COVID-19 pneumonia	74.58	99.46	88.79	0.98		
	Other pneumonia	97.42	84.93	88.38	0.97		
	Nonpneumonia	100	99.42	99.59	0.99		

^a^AUC: area under the curve.

## Discussion

### Principal Findings

We were able to develop the FCONet deep learning models to diagnose COVID-19 pneumonia in a few weeks using transfer learning based on pretrained models. The FCONet based on ResNet-50 showed excellent diagnostic performance to detect COVID-19 pneumonia. Although the diagnostic accuracy of the FCONet models based on VGG16, ResNet-50, and Xception was excellent in the testing data set (sensitivity, 97.91%, 100%, and 97.91%, respectively; specificity, 100%, 99.64% and 99.29%, respectively), external validation using the low-quality image data set demonstrated that detection accuracy was the highest with ResNet-50 (96.97%), followed by Xception (90.71%), Inception-v3 (89.38%), and VGG16 (87.12%).

To collect as many images as possible within a limited time, we collected readily available chest CT images of COVID-19 patients from institutions in our region (WKUH and CNUH) and a public COVID-19 database established by SIRM. We also systematically searched for chest CT images of COVID-19 embedded in recent papers published between January 19 and March 25, 2020. As these CT images in the published paper were of low quality, we used them only in an additional external validation data set.

During a national crisis such as the COVID-19 pandemic, when the number of infected patients is precipitously increasing and physicians are occupied combating the disease, rapid development of AI methods to detect COVID-19 in CT is crucial to alleviate the clinical burden of physicians and to increase the efficiency of the patient management process [8]. However, significant challenges remain when developing such AI techniques within a limited time to collect CT data and train AI models.

To save time for AI training, we used the chest CT images directly without preprocessing of the lung segmentation. In general, lung segmentation preprocessing is regarded to improve the accuracy of AI training [24-27]; we believe that this improvement can be traded off in exchange for saving time. For AI training, we chose the transfer learning algorithms. Transfer learning enabled us to save time by using pretrained CNN models in the ImageNet data sets, including VGG16, ResNet-50, Inception-v3, and Xception [28]. In our study, FCONet based on ResNet-50 showed excellent results and outperformed the FCONet models based on the other three pretrained models in both our testing data set and the additional external validation data set. The VGG model is regarded as a traditional sequential network architecture and may be hampered by slow training and a large model size [17]. The ResNet-50 model is characterized by network-in-network architectures, which have much deeper layers than those of VGG models, enabling reduction of the model size [18]. Our results suggest that transfer learning for a 2D deep learning framework can be robustly applied to deep learning models and that the ResNet-50 model provides the best accuracy.

We adopted AI training based on a 2D image framework rather than a 3D framework because 3D deep learning requires significantly higher computation power than sequential 2D image analyses [29]. In our emergent clinical setting to fight COVID-19, a simple and rapid model may be preferable to a complex and slow model. In addition, training a 2D image framework saves time for AI development.

Despite limited resources and time, we were able to generate a deep learning model to detect COVID-19 from chest CT with excellent diagnostic accuracy. To date, a few papers have been published on AI models for detecting COVID-19 in chest CT images [6]. An AI model named COVNet was trained using 4356 CT images from six hospitals in China. It showed 90% sensitivity (95% CI 83%-94%) and 96% specificity (95% CI 93%-98%) in detecting COVID-19, which is comparable with our results. However, we cannot compare our FCONet to COVNet because the training and testing data sets are different.

Although chest radiography is the most commonly used imaging tool to detect COVID-19, its sensitivity is lower than that of CT [30]. However, in this pandemic period, clinicians may hesitate to perform chest CT due to limited resources such as CT scanners and radiologists as well as contamination of CT scanners [31]. In our hospitals (WKUH and CNUH), we recently dedicated a mobile CT scanner exclusively to COVID-19 patients to alleviate the physical and mental stress of medical staff. We believe that incorporating an AI model to detect suspicious lesions of COVID-19 pneumonia can improve the workflow by providing rapid diagnostic support.

### Limitations and Future Work

Our study has several limitations. Firstly, our AI models were validated mainly using a split testing data set. Thus, the testing data set was obtained from the same sources as the training data set. This may raise issues of generalizability and overfitting of our models [32,33]. Indeed, the detection accuracy of our model decreased slightly for the external validation data set using chest CT images from published papers. However, the initial goal was to incorporate a deep learning model in our emergent clinical setting as a supporting tool. In the near future, we will train our model using CT images from various institutions and countries. Secondly, we used a relatively small amount of data to train the deep learning models. Thus, we will establish a sustainable AI training system that can continue to train our model using prospectively collected CT images.

### Conclusions

We described FCONet, a simple 2D deep learning framework based on a single chest CT image, as a diagnostic aid that provides excellent diagnostic performance to diagnose COVID-19 pneumonia. The FCONet model based on ResNet-50 appears to be the best model, outperforming other models based on VGG16, Xception, and Inception-v3.

## Abbreviations

AI: artificial intelligence
ANOVA: analysis of variance
API: application programming interface
AUC: area under the curve
CNN: convolutional neural networks
CNUH: Chonnam National University Hospital
COVID-19: coronavirus disease
CT: computed tomography
DICOM: Digital Imaging and Communications in Medicine
FCONet: fast-track COVID-19 classification network
FN: false negative
FP: false positive
Grad-CAM: gradient-weighted class activation mapping
ROC: receiver operating characteristic
SARS-CoV-2: severe acute respiratory syndrome coronavirus 2
SIRM: Italian Society of Medical and Interventional Radiology
TN: true negative
TP: true positive
WKUH: Wonkwang University Hospital
